# Analysis of the gut microbiota of walking sticks (Phasmatodea)

**DOI:** 10.1186/1756-0500-6-368

**Published:** 2013-09-11

**Authors:** Matan Shelomi, Wen-Sui Lo, Lynn S Kimsey, Chih-Horng Kuo

**Affiliations:** 1 Department of Entomology, University of California, Davis, USA; 2 Institute of Plant and Microbial Biology, Academia Sinica, Taipei, Taiwan; 3 Molecular and Biological Agricultural Sciences Program, Taiwan International Graduate Program, National Chung Hsing University and Academia Sinica, Taipei, Taiwan; 4 Graduate Institute of Biotechnology, National Chung Hsing University, Taichung, Taiwan; 5 Biotechnology Center, National Chung Hsing University, Taichung, Taiwan

**Keywords:** Phasmatodea, Microbiota, 16S rDNA, Symbionts, Digestive system

## Abstract

**Background:**

Little is known about the Phasmatodea gut microbial community, including whether phasmids have symbiotic bacteria aiding in their digestion. While symbionts are near ubiquitous in herbivorous insects, the Phasmatodea’s distinctively thin body shape precludes the gut enlargements needed for microbial fermentation. High-throughput sequencing was used to characterize the entire microbiota of the fat bodies, salivary glands, and anterior and posterior midguts of two species of walking stick.

**Results:**

Most bacterial sequences belonged to a strain of *Spiroplasma* (Tenericutes) found primarily in the posterior midgut of the parthenogenetic species *Ramulus artemis* (Phasmatidae). Beyond this, no significant differences were found between the *R*. *artemis* midgut sections or between that species and *Peruphasma schultei* (Pseudophasmatidae). Histological analysis further indicated a lack of bacteriocytes.

**Conclusions:**

Phasmids are unlikely to depend on bacteria for digestion, suggesting they produce enzymes endogenously that most other herbivorous insects obtain from symbionts. This conclusion matches predictions based on phasmid anatomy. The role of *Spiroplasma* in insects warrants further study.

## Background

Research on insect endosymbionts has historically focused on insects with limited diets, such as wood-feeders or the nitrogen-limited phloem feeders [1,2]. This body of work has revealed several obligate symbioses, such as the aphid-*Buchnera* system where the insect cannot survive without its bacterial mutualist [3], as well as the many termite symbioses with gut bacteria and/or flagellates that assist in lignocellulose digestion [4]. Many such insects have specialized cells called bacteriocytes in which the microbes are housed [5], while in others the symbionts persist within the midgut lining [2]. As most microbes, including the majority of endocellular symbionts, cannot be cultured [6], high-throughput sequencing has been used to characterize insect microbiota and identify possible symbionts [7], with successes in groups such as honey bees [8] and moths [9]. Gut microbes and their enzymes have received much recent attention by the biofuel industry’s search for novel cellulases and xylanases, which has biased the gut microbe literature towards xylophages like termites, roaches, and beetles [10,11].

Less understood are the digestive mechanisms and gut microbes of leaf eaters such as Lepidoptera larvae and orthopteroids. While leaves are not as difficult a diet as dry wood, they still contain abundant cellulase as plant cell walls [12], as well as toxic secondary chemicals, waxes, trichomes, and other obstacles to consumption and digestion by insects [13]. Symbiotic microbes would be beneficial to leaf eaters by assisting in cellulose breakdown [14], nitrogen fixation or amino-acid metabolism [15], detoxifying or neutralizing plant defensive compounds [16], and recycling nitrogenous wastes [17].

**Figure 1:**
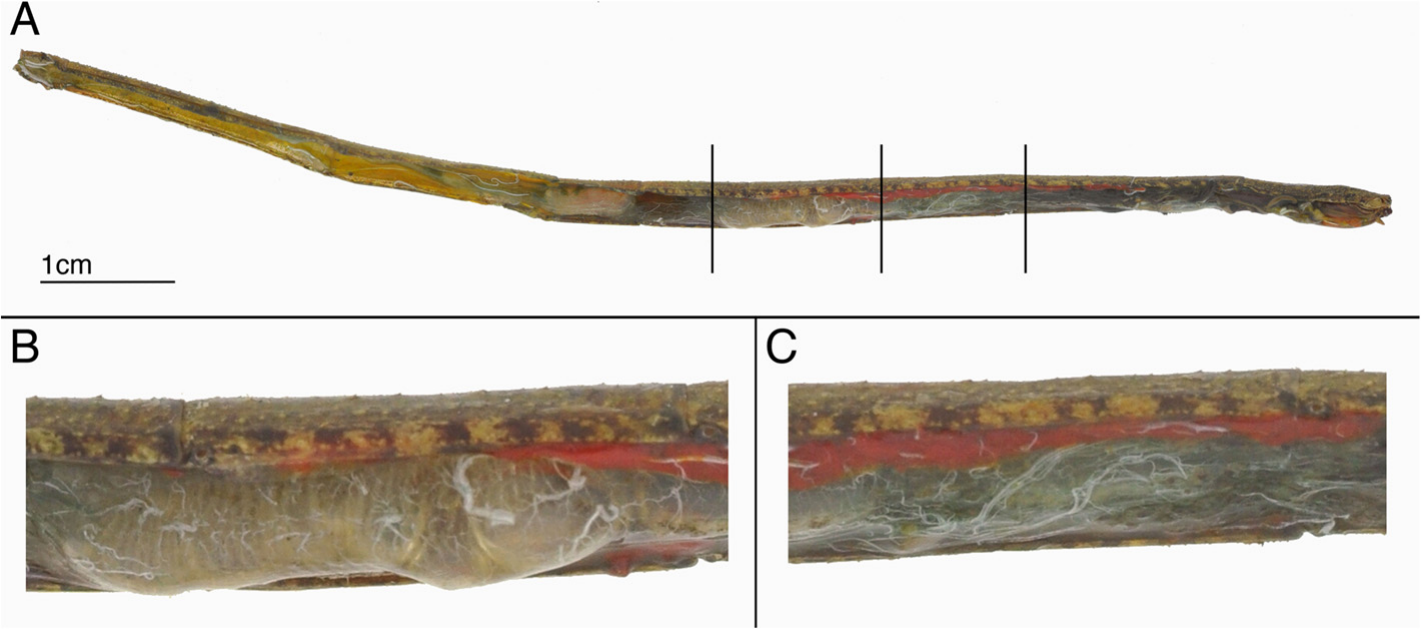
**The digestive tract within a partially**-**dissected adult female *Ramulus artemis. *(A)** Whole gut in body cavity, minus head. Inserts are close-ups of the **(B)** anterior and **(C)** posterior midguts.

While some microbial work has been performed on Orthoptera *sensu stricto*[18-20], and a few papers have looked at specific microbes within phasmids [21,22], no complete microbial inventory of the Phasmatodea gut has ever been performed. As obligate leaf eaters (as opposed to certain periodically cannibalistic orthopteroids), the phasmids would certainly stand to benefit from having microbial symbionts. However, several factors suggest that phasmids would not have digestive symbionts. Their characteristic body shape places restrictions on their gut morphology, which is straight and narrow tubes (Figure 1) with very short gastric caecae and no obvious diverticulae or fermentation chambers [23,24] of the kind that house microbes in other insects [1]. The only likely places for a phasmid symbiont to exist would be the midgut (the main site of phasmid digestion), the salivary glands, and the fat bodies. One paper studying insect fibre digestion suggested phasmids do not rely on microbes for digestion, but it based this on counts of culturable microbes, meaning up to 99% of the microbe diversity in the gut may have been ignored [21]. In our paper, microscopy and next generation sequencing was used to catalog the entire microbiota of the aforementioned phasmid organs from two species, with the goal of finding potential symbionts and possibly elucidating their functions, as well as increasing our knowledge of a poorly described alimentary canal.

## Methods

The phasmids used were lab-reared specimens of *Ramulus artemis* (Westwood) (Phasmatidae), fed rose (*Rosa*) leaves, and *Peruphasma schultei* (Conle & Hennemann) (Pseudophasmatidae), fed privet (*Ligustrum*). All insects were maintained and used as per the University of California, Davis’ Institutional Animal Care and Use Committee guidelines.

To check for the presence of bacteriocytes, histological analysis was performed [25]. Whole insects with longitudinal slits across the body wall were fixed in Bouin’s Fluid for three days and stored in 70% ethanol. Tissue samples were dissected out, dehydrated in an ethanol-butanol series, and embedded in paraffin. Sections were slide mounted using Meyer’s albumin and stained in Giemsa or Heidenhaim’s haematoxylin and eosin for light microscopy [26,27].

For molecular analysis, adult females were starved for two days to clear their guts and reduce chloroplast contamination of the samples. The digestive tract and its contents were dissected out, and the midguts divided into two sections reflecting the phasmid’s unique gut anatomy (Figure 1): the heavily pleated anterior midgut (AMG) and the unpleated posterior midgut (PMG) studded with tubules of a currently unknown function [23,28]. The gut sections as well as the fat bodies and salivary glands were preserved in 100% ethanol prior to DNA extraction.

The total DNA from each sample was extracted using the Wizard® Genomic DNA Purification Kit (Promega; Fitchburg, Wisconsin, USA) following the manufacturer’s protocol. To identify gut microbes, PCR was done to amplify the 16S ribosomal DNA using the universal primers 27F (AGAGTTTGATCMTGGCTCAG) and 511R (GCGGCTGCTGGCACRKAGT) with the appropriate 454 Life Sciences adaptor sequence. In addition, the forward primer used for each sample contained a unique 6-bp barcode for multiplexed sequencing. The barcodes used are described in NCBI BioSamples SAMN02318746-SAMN02318757. To minimize biases that might occur in individual PCR reactions, three independent reactions were performed for each sample and the products were pooled before sequencing. Each of the 50 μL of PCR mixture consisted of 1 μL PfuUltra II Fusion HS DNA polymerase (Stratagene; La Jolla, California, USA), 5 μL of supplied 10× buffer, 2.5 μL of 5 mM dNTP mix (MBI Fermentas; Burlington, Ontario, Canada), 0.5 μL of 10 mg/mL BSA (New England Biolabs; Ipswich, Massachusetts, USA), 1 μL of each 10 μM primer, and 50 ng of template DNA. The PCR program included one denaturing step at 95°C for 3 min, 25 cycles of 95°C for 40 sec, 55°C for 40 sec, and 72°C for 40 sec, followed by a final extension at 72°C for 7 min. Gel electrophoresis was used to check the existence of a single band of expected size for each PCR product. For the positive samples, PCR products were purified with the QIAquick PCR Purification Kit (Qiagen; Venlo, Netherlands). To further confirm the successful amplification of bacterial 16S rDNA in our broad range PCR, the purified PCR products were cloned using the CloneJet PCR Cloning Kit (Fermentas Life Science; Burlington, Ontario, Canada) and transformed into HIT-JM 109 competent cells (RBC Bioscience; Zhonghe City, Taipei County, Taiwan). A limited number of clones were sequenced using the BigDye Terminator v3.1 Cycle Sequencing Kit on an ABI Prism 3700 Genetic Analyzer (Applied Biosystems; Foster City, California, USA) to verify the presence of expected 16S rDNA fragment, multiplexing barcodes, and the adapters for 454 sequencing.

For high-throughput DNA sequencing, the positive samples were pooled in equal proportions and sequenced on a 454 Jr. sequencer (454 Life Sciences; Branford, Connecticut, USA). The pyrosequencing flowgrams were converted to sequence reads with corresponding quality scores using the standard software provided by 454 Life Sciences. All raw reads were deposited in NCBI Sequence Read Archive (accession number SRR955712). The sequences were quality-trimmed using the default settings of LUCY [29]. Reads that were shorter than 400-bp after the quality trimming were removed from the data set. After the quality trimming, the sample-specific barcode and the primer sequence were identified and trimmed from each sequence; sequences that lacked a recognizable barcode and PCR primer were discarded. To identify the operational taxonomic units (OTUs) in these samples, the partial 16S rDNA sequences were hierarchically clustered at 100%, 99%, and 97% sequence identity using USEARCH version 5.2.32 [30]. The 97% sequence identity threshold was chosen because it is commonly used to define bacterial species [31,32]. For taxonomic assignment, the representative sequence of each OTU was used as the query for the CLASSIFIER [33] program provided by the Ribosomal Database Project [34] with the 16S rRNA training set (version 2.5). The OTUs that were identified as originating from plant chloroplasts or mitochondria were excluded from downstream analyses. Furthermore, OTUs that could not be assigned to a particular genus with at least 70% confidence level were removed because these sequences are likely to represent chimeras or other artifacts introduced during the PCR or pyrosequencing process [35]. For verification of the CLASSIFIER results and taxonomic assignment at species level, BLASTN [36,37] similarity search against the NCBI nt database [38] was performed for the representative sequence of each OTU. We limited the BLASTN search to the subject sequences from Bacteria (‘taxid2’). Additionally, subject sequences from environmental samples or metagenomes were excluded because these sequences often do not contain reliable taxonomic assignments. The e-value cutoff and other parameters of the BLASTN search were set to default; the top one hit was used as the representative for each query.

To compare the microbiota composition among individual samples, we utilized the software package Fast UniFrac [39] to perform Principal Coordinate Analysis (PCoA) and hierarchical clustering. The OTUs were weighted by abundance and the branch lengths were normalized. To generate a reference tree for the Fast UniFrac analyses, the representative sequences from all OTUs were aligned using the RDP ALIGNER [34]. The resulting multiple sequence alignment was examined to ensure that the 5’-end of each sequence was mapping to the expected location of 16S rDNA. The program FastTree [40] was then used to infer a maximum likelihood phylogeny of the OTUs. Additionally, the PVCLUST package [41] for R Statistical Software [42] was used to perform an alternative hierarchical clustering analysis with a phylogeny-independent approach.

## Results

**Figure 2:**
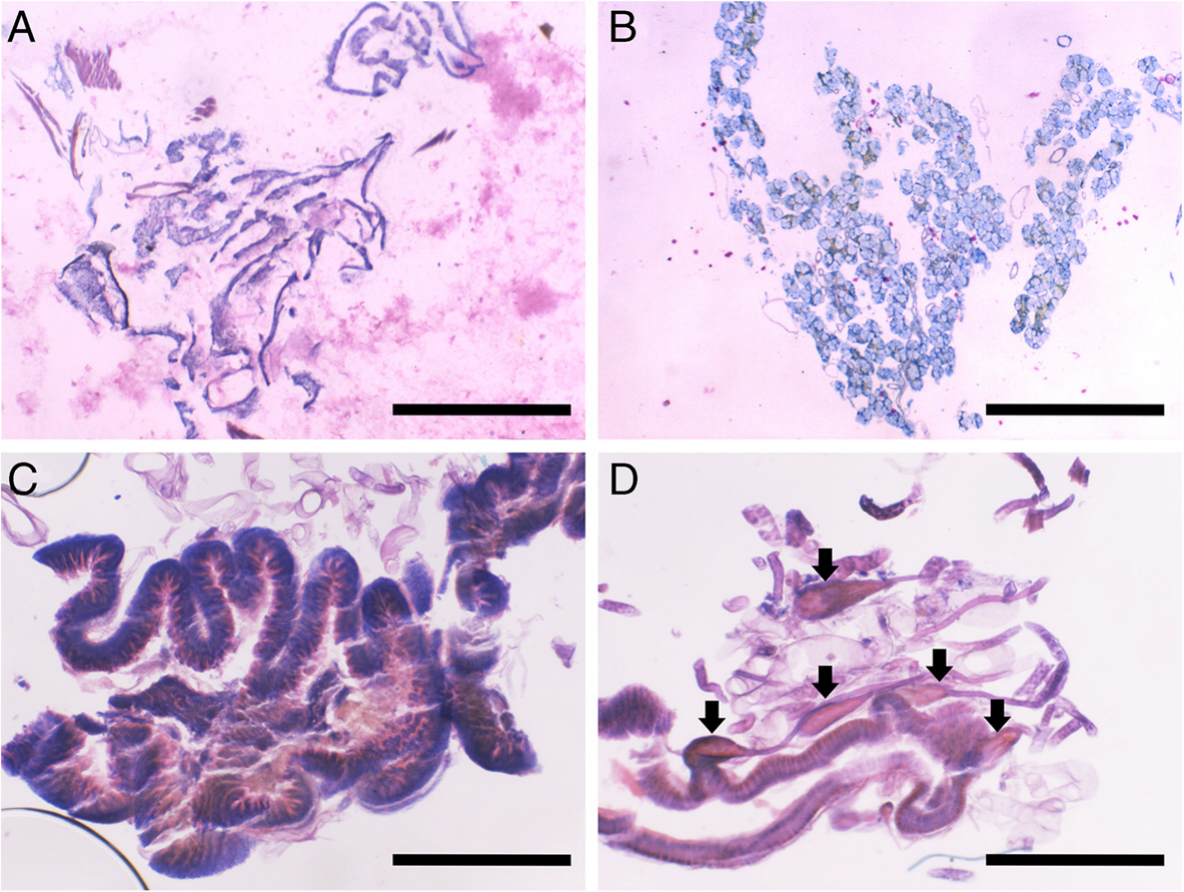
**Histological sections from a late instar male *Peruphasma schultei, stained in Giemsa solution.*** Scale bars for all are 1 mm in length. The images shown here are exemplary of all observed sections. **(A)** Fat body. **(B)** Salivary Glands. **(C)** Anterior midgut, with notable pleating. **(D)** Posterior midgut, unpleated and studded with ampullae-tubules of unknown function (labeled with arrows).

The broad range PCR amplification results for bacterial 16S rDNA were negative for the fat body and salivary gland samples examined, so they were excluded after the initial quality check steps. This negative result is consistent with phasmid histological studies, which did not show obvious mycetocytes in fat body or salivary gland tissue, nor any obvious endosymbionts in the midgut tissues (Figure 2). All seven *P*. *schultei* PMG samples tested negative for bacterial DNA as well. In total, bacterial 16S rDNA was recovered from four *P*. *schultei* AMGs, four *R*. *artemis* AMGs, and three *R*. *artemis* PMGs, and these samples were included in the 454 sequencing and final analysis. In total, we obtained 26,006 high quality sequence reads for these 11 midgut samples.

**Figure 3:**
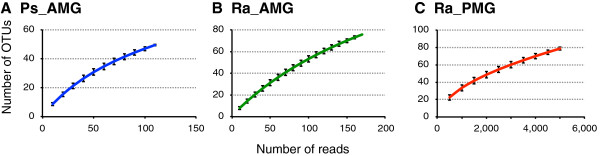
**Rarefaction curves of the three sample types.** The individual samples of the same type were pooled and resampled 10,000 times to determine the number of OTUs found with different numbers of sequencing reads. The error bars indicate ± one standard deviation. **(A)***P*. *schultei* anterior midgut (Ps_AMG) samples. **(B)***R*. *artemis* anterior midgut (Ra_AMG) samples. **(C)***R*. *artemis* posterior midgut (Ra_PMG) samples.

**Figure 4:**
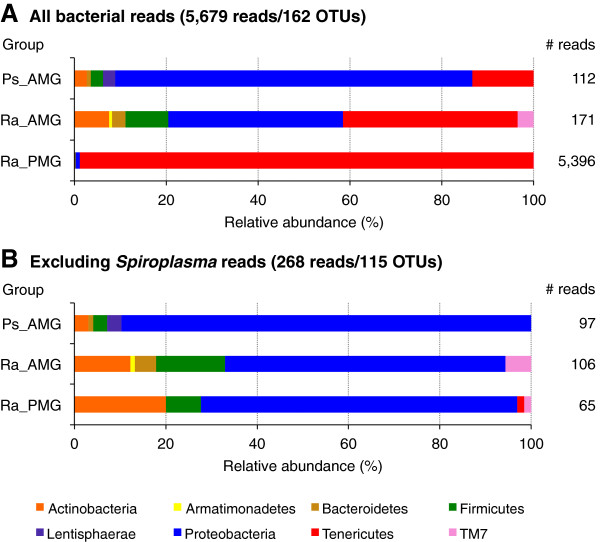
**Relative abundances of reads of the different phyla. (A)** All 5,679 bacterial 16S rDNA reads. **(B)** Excluding *Spiroplasma* reads.

Among the midgut samples, an overwhelming majority of the sequencing reads originated from the 16S rDNA of plant chloroplasts or mitochondria (Table 1). After removing these contaminants, we obtained a total of 162 bacterial OTUs covering 64 genera (Table 2). Rarefaction curves (Figure 3) suggest that the sampling depths achieved in this study may not be sufficient to fully characterize the microbiota of Phasmatodea guts. With the exception of a large Tenericute population in the *R*. *artemis* PMGs, most of the microbes isolated were Proteobacteria, followed by Actinobacteria and Firmicutes (Table 1 and Figure 4). Nearly all of the Tenericute reads (4,902 out of 5,412 Tenericute sequences) belonged to a single OTU (OTU_ID: CXTIA; Additional file 1) found mostly in the *R*. *artemis* posterior midgut. The BLASTN search against the NCBI nucleotide database showed that this sequence is 99.0% identical to *Spiroplasma* sp. ‘Gent’ (GenBank AY569829), an uncultured, male-killing entomopathogen identified from the housefly *Fannia manicata*. The next most common OTU had only 161 reads, and all other OTUs had less than 50 reads (Additional file 1). All but one of the 10 most abundant OTUs appeared to be *Spiroplasma*, which are known to be associated with a wide range of insects [43,44]. The most abundant non-*Spiroplasma* OTU was represented by 31 reads and showed high similarity (99.3% sequence identity) to *Sphingobium sp*. KR5 (GenBank JQ433940).

**Table 1:** Summary of reads received from phasmid samples

	***P. schultei *anterior midgut**	***R. artemis *anterior midgut**	***R. artemis *posterior midgut**	**Total**
# of positive samples	4	4	3	
**All sequences:**				
# reads passed quality control	9,255	9,697	7,054	26,006
Average # reads per sample	2,313.75	2,424.25	2,351.33	
# 100% id OTUs				8,090
# 99% id OTUs				2,702
# 97% id OTUs				844
**After RDP CLASSIFIER:**				
# chloroplast 16S				14,541
# mitochondrial 16S and other contaminants				5,786
# bacterial 16S reads	112	171	5,396	5,679
Average # reads per sample	28	43	1,799	
# reads (# OTUs) by phylum				
Actinobacteria	3(2)	13(9)	13(4)	29(13)
Armatimonadetes	0	1(1)	0	1(1)
Bacteroidetes	1(1)	5(3)	0	6(4)
Firmicutes	3(3)	16(9)	5(4)	24(15)
Lentisphaerae	3(1)	0	0	3(1)
Proteobacteria	87(37)	65(39)	45(26)	197(75)
Tenericutes	15(6)	65(10)	5,332(47)	5,412(47)
TM7	0	6(5)	1(1)	7(6)

Phyla Based on RDP CLASSIFIER results.

**Table 2:** **The number of reads for each microbe genus per gut type** (**pooled samples**)

**Phylum**	**Genus**	***Ps *AMG**	***Ra *AMG**	***Ra *PMG**	**Total**
Actinobacteria	*Arthrobacter*		3		3
Actinobacteria	*Brevibacterium*	1	1	2	4
Actinobacteria	*Conexibacter*		2		2
Actinobacteria	*Janibacter*		2		2
Actinobacteria	*Propionibacterium*	2	3	11	16
Actinobacteria	*Sanguibacter*		1		1
Actinobacteria	*Streptomyces*		1		1
Armatimonadetes	Armatimonadetes_gp5		1		1
Bacteroidetes	*Bacteroides*	1			1
Bacteroidetes	*Cloacibacterium*		4		4
Bacteroidetes	*Flavobacterium*		1		1
Firmicutes	*Bacillus*		3	1	4
Firmicutes	*Clostridium* sensu stricto			1	1
Firmicutes	*Clostridium* XI	1			1
Firmicutes	*Exiguobacterium*		1		1
Firmicutes	*Lactococcus*		1		1
Firmicutes	*Pelospora*		2		2
Firmicutes	*Planifilum*			2	2
Firmicutes	Planococcaceae_incertae_sedis			1	1
Firmicutes	*Sedimentibacter*	1			1
Firmicutes	*Staphylococcus*	1	6		7
Firmicutes	*Streptococcus*		2		2
Firmicutes	*Weissella*		1		1
Lentisphaerae	*Victivallis*	3			3
Proteobacteria	*Acidovorax*	4	4	4	12
Proteobacteria	*Acinetobacter*	7	2	4	13
Proteobacteria	*Alcanivorax*			1	1
Proteobacteria	*Aquabacterium*	10	4	6	20
Proteobacteria	*Arcobacter*	10	4	3	17
Proteobacteria	*Azospira*			1	1
Proteobacteria	*Belnapia*		1		1
Proteobacteria	*Bordetella*		1		1
Proteobacteria	*Brevundimonas*	2	1		3
Proteobacteria	*Burkholderia*		3		3
Proteobacteria	*Caulobacter*	1	3		4
Proteobacteria	*Comamonas*	1	1	1	3
Proteobacteria	*Cupriavidus*	2		2	4
Proteobacteria	*Enhydrobacter*	2			2
Proteobacteria	*Escherichia*/*Shigella*	1		1	2
Proteobacteria	*Herbaspirillum*		2	1	3
Proteobacteria	*Hyphomicrobium*	1			1
Proteobacteria	*Legionella*	3			3
Proteobacteria	*Methylobacterium*	2	2		4
Proteobacteria	*Methyloversatilis*		2	1	3
Proteobacteria	*Novosphingobium*	5	2		7
Proteobacteria	*Ochrobactrum*	1	2		3
Proteobacteria	*Pantoea*		3		3
Proteobacteria	*Paracoccus*		2		2
Proteobacteria	*Pelomonas*	1			1
Proteobacteria	*Pseudomonas*	4	8	4	16
Proteobacteria	*Raoultella*			1	1
Proteobacteria	*Rheinheimera*			1	1
Proteobacteria	*Rhizobacter*		4		4
Proteobacteria	*Shewanella*		1		1
Proteobacteria	*Simplicispira*		4		4
Proteobacteria	*Smithella*	1	1		2
Proteobacteria	*Sphingobium*	21	6	9	36
Proteobacteria	*Sphingomonas*	4	1	2	7
Proteobacteria	*Stenotrophomonas*		1		1
Proteobacteria	*Undibacterium*	1		2	3
Proteobacteria	*Zoogloea*	3		1	4
Tenericutes	*Haloplasma*			1	1
Tenericutes	*Spiroplasma*	15	65	5,331	5,411
TM7	TM7_genera_incertae_sedis		6	1	7
Sum		112	171	5,396	5,679

The taxomic assignment at genus level was based on RDP CLASSIFIER. *Ps* = *P*. *schultei*. *Ra* = *R*. *artemis*.

**Figure 5:**
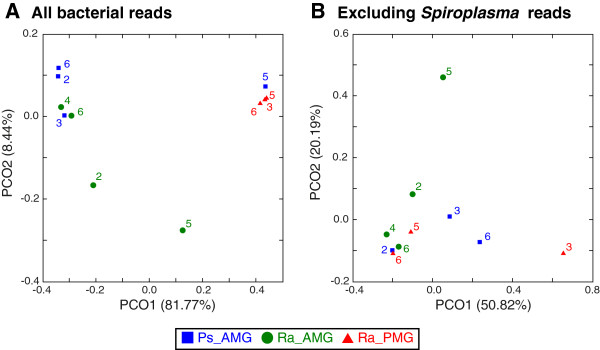
**Principal Coordinate Analyses (PCoA) of the microbiota.** The samples are color-coded by type: blue squares, *P*. *schultei* anterior midgut (Ps_AMG) samples; green circles, *R*. *artemis* anterior midgut (Ra_AMG) samples; red triangles, *R*. *artemis* posterior midgut (Ra_PMG) samples. The label indicates the individual id within each species. The numbers in parentheses in axis labels indicate the percentage of variance explained. **(A)** All 5,679 bacterial 16S rDNA reads. **(B)** Excluding *Spiroplasma* reads.

Comparison of the microbiota composition based on the PCoA plots (Figure 5) indicated that the relative abundance of *Spiroplasma* reads is the major determinant that differentiates different sample types. When all 5,679 bacterial reads are considered, the three *R*. *artemis* PMG samples form a tight cluster and the PCO1 explains 81.77% of the variance (Figure 5A). Although one *P*. *schultei* AMG sample (from individual #5) appears to share a similar microbiota composition with the three *R*. *artemis* PMG samples, this *P*. *schultei* AMG sample should be considered as an outlier because it contains only one reads assigned to the most abundant *Spiroplasma* OTU. When the putative *Spiroplasma* reads were excluded, no clear pattern exists to distinguish among sample types (Figure 5B).

**Figure 6:**
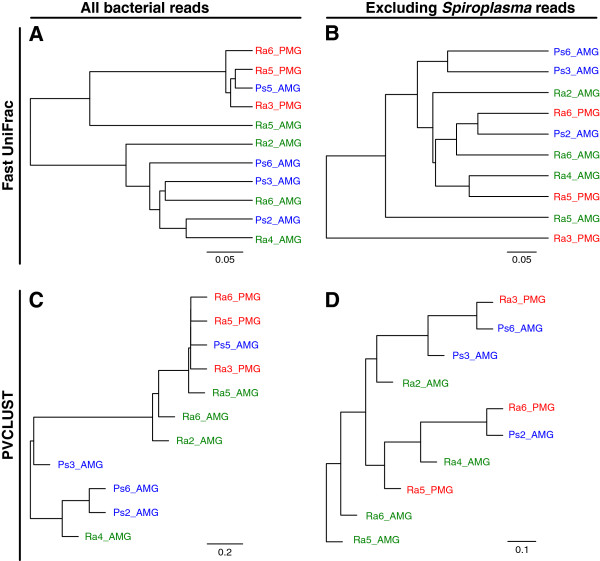
**Hierarchical clustering analysis.** Labels for sample ids: Ps#_AMG = *P*. *schultei* anterior midgut, Ra#_AMG = *R*. *artemis* anterior midgut, Ra#_PMG = *R*. *artemis* posterior midgut; the number indicates the individual id within each species (e.g., Ra6_AMG and Ra6_PMG represent the anterior midgut and the posterior midgut of individual #6 from *R*. *artemis*). Panels **(A)** and **(B)**: Phylogeny-dependent clustering based on Fast UniFrac; Panels **(C)** and **(D)**: Phylogeny-independent clustering based on PVCLUST.

The hierarchical clustering analyses based on either phylogeny-dependent approach (Figure 6 panels A and B) or phylogeny-independent approach (Figure 6 panels C and D) produced the same patterns inferred from the PCoA plots. When all bacterial reads are considered (Figure 6 panels A and C), the three *R*. *artemis* PMG samples (together with the Ps5_AMG sample that contains only one *Spiroplasma* read) form a single clade with short distances among each other and high divergences with other samples. When the *Spiroplasma* reads were excluded (Figure 6 panels B and D), individual samples of the same type do not cluster together. Additionally, the branches that separate samples of the same type are similar in length compared to those separating samples of different types.

## Discussion

The overabundance of chloroplast contamination greatly reduced the amount of bacterial reads available for analysis. Starving the phasmids for two days was insufficient to clear their digestive tracts. For future study, a longer time period of starvation (e.g., five to ten days) would be preferable. While 16S primers that select against chloroplasts do exist, they also bias the results of broad range PCR and are not suitable replacements for “universal” primers when studying total microbial community. Despite the relatively low abundance of bacterial reads, the use of next generation sequencing techniques still provided thousands of usable reads of mostly uncultivated microbes. This data set provides at least one order of magnitude more reads than would be available using clone libraries or denaturing gradient gel electrophoresis.

The low number of bacterial reads compared to chloroplast reads suggests the abundance of bacterial cells in the phasmid gut is relatively low. These results, together with the lack of bacterial DNA in the fat body and salivary glands and the lack of bacteriocytes in the tissue slices, do not support the hypothesis that phasmids have obligate microbial symbioses. Had such mutualist endosymbionts existed in these samples, they would have been detected at similar levels to the *Spiroplasma* infestation. While lab-reared phasmids will likely have different gut microbiota from wild specimens, they are expected to retain any symbiotic microbes essential for survival. Symbiont absence in the lab suggests absence in the field as well, but this remains to be demonstrated. The data suggests that most of the microbes isolated were environmental, picked up by the phasmids from their diet or housing [18,45].

The lack of beneficial symbiotic microbes in phasmids is unsurprising given the aforementioned restrictions on their digestive system due to their distinct body shape. The phasmid gut lacks the space to develop the significantly modified sections seen in symbiont-housing organisms, such as the enlarged hindguts of termites. Given these restrictions, one would expect a phasmid to compensate for lack of symbionts either with modified feeding behavior (such as increased consumption and/or slower gut transit time) or through endogenous production of digestive enzymes such as cellulases, which research predicts the phasmids can produce [12,21,46]. No research on phasmid enzymes has been published yet, however. If phasmids did depend on gut bacteria to breakdown their leafy diet, one would expect a gut microbiota consisting predominantly of Bacteroidetes and Firmicutes, as one sees in the roaches and termites [2]. The abundance of Proteobacteria resembles the mostly transient microbes found in Orthoptera [18], which supports the closer phylogenetic relationship between that order and the Phasmatodea [47].

The nature of *R*. *artemis*’ *Spiroplasma* infestation needs investigation. Of the 162 OTUs, 32 were closely related to *Spiroplasma* sp. ‘Gent’ (GenBank AY569829), and another eight were closely related to *Spiroplasma ixodetes* (GenBank GU585671). The infection’s localization to the PMG suggests the bacteria may be colonizing the PMG’s enigmatic appendices, but histological data did not confirm this (Figure 2D). *Spiroplasma* infestation may also explain why *R*. *artemis* can be parthenogenetic in cultures, as some *Spiroplasma* species are known male-killing parasites [48] similar to *Wolbachia*[49]. Several other phasmid species, most notably the laboratory stick insect, *Carausius morosus*, are also parthenogenetic [50], so checking these cultures for *Spiroplasma* infestations may be revealing. DiBlasi et al. [22] concluded that the *Spiroplasma* is transmitted in phasmids by mites, yet the cultures used here were mite-free, suggesting vector-less transmission.

The most common non-*Spiroplasma* microbe isolated was identified as a member of *Sphingobium*, which may be involved in the degradation of aromatic hydrocarbons [51]. It may perform this role for the phasmid, aiding in digestion by degrading plant defensive compounds, but whether it is a true symbiont transmitted between individuals, an environmental microbe that can colonize the gut, or an allochthonous (transient) microbe found on the leaves and just passing through is unknown.

## Conclusions

This paper marks the first attempt to catalog the microbial diversity of the Phasmatodea (Additional file 1: Table S1). While a clade of symbiont-dependent phasmids may exist, the likelihood is low. All evidence suggests the only heritable symbionts, allegedly ubiquitous in the Insecta [6], in the Phasmatodea are reproduction-manipulators like *Spiroplasma*. Still, the phasmid gut microbial community is diverse and merits further investigation. The possibility of fungal or other eukaryotic symbionts in the gut remains. The microbial community may function together to benefit the phasmid in ways akin to their functions on leaf surfaces, such as secondary chemical detoxification [52]. Lastly, if phasmid digestion is truly microbe-independent, then the enzymology of the gut demands further analysis given the potential for finding novel lignocellulases and other compounds of possible human industrial applications.

### Availability of supporting data

A supplementary table (Table S1) the lists the taxonomic assignment OTUs is included as Additional file 1.

## Competing interests

The authors declare that they have no competing interests.

## Authors’ contributions

MS and CHK conceived of the study. MS and WSL carried out the experiments. MS and CHK performed the data analysis. MS, LSK, and CHK wrote the manuscript. All authors read and approved the final manuscript.

## Supplementary Material

Additional file 1: Table S1 Taxonomic assignment of the OTUs based on RDP CLASSIFIER and BLASTN sequence similarity search against the NCBI nt database. Click here for file
